# *In vitro* evaluation of CD40-targeting aptamer-based DNA vaccine adjuvants using HD11 cell culture

**DOI:** 10.3389/fvets.2026.1735450

**Published:** 2026-03-30

**Authors:** Abigeal I. Omolewu, Santiago U. Diaz, Seong W. Kang, Christine N. Vuong, Adil Al-Ogaili, Billy M. Hargis, Young Min Kwon

**Affiliations:** 1 Department of Poultry Science, University of Arkansas System Division of Agriculture, Fayetteville, AR, United States; 2 Department of Medical Laboratory Techniques, Kut Institute of Technology, Middle Technical University, Baghdad, Iraq; 3 Cell and Molecular Biology Program, University of Arkansas, Fayetteville, AR, United States

**Keywords:** CD40-targeting, chicken, DNA aptamer, rolling circle amplification, *Salmonella*, vaccine adjuvant

## Abstract

*Salmonella* is a major foodborne pathogen that colonizes the gastrointestinal tract of poultry, compromising bird health and productivity while posing significant public health threats. Consumption of contaminated poultry products is the leading cause of *Salmonella*-related illnesses in humans. Although numerous vaccination strategies exist, weak immune responses often limit the efficacy. One promising approach to address this limitation is targeting the cluster of Differentiation 40 (CD40), a costimulatory receptor expressed on antigen-presenting cells that plays a crucial role in initiating and regulating immune response. In this study, we developed two rolling circle amplification (RCA) vaccine complexes (RCA_v3 and RCA_v5) against *Salmonella*, each comprising a CD40-targeting component to enhance immune activation (Aptamer RCA II) and a *Salmonella*-binding component for antigen delivery (Anti-*Salmonella* RCA). We evaluated the two RCA vaccine complex designs for their ability to activate macrophages in the HD11 chicken cell line by quantifying nitric oxide production via the Griess assay. The results showed that the two RCA vaccine designs (RCA_v3 and RCA_v5) combined with formalin-killed *Salmonella* Enteritidis (SE) significantly (58.00 μM and 52.08 μM, respectively) enhanced macrophage activation (*p* < 0.05) compared to SE alone (33.37 μM). Also, the activation levels of RCA_v3 vaccine complex were significantly higher with SE as compared with the respective counterparts without SE, RCA_v3 alone, and Aptamer RCA II (35.13 μM and 39.02 μM, respectively) (*p* < 0.05). These findings offer valuable insights into developing effective vaccination strategies to control *Salmonella* colonization in poultry, presenting a promising approach to improving food safety and reducing public health risks associated with *Salmonella* infections in chickens.

## Introduction

*Salmonella,* a gram-negative, rod-shaped, facultatively anaerobic bacterium in the Enterobacteriaceae family, is a zoonotic pathogen responsible for human gastrointestinal disease. Contaminated poultry products, particularly meat and eggs, are the primary source of *Salmonella* infection in humans (1). Nontyphoidal *S. enterica* serovars, such as Typhimurium and Enteritidis, can infect multiple host species, complicating their transmission dynamics. Although these serotypes rarely cause noticeable symptoms in poultry, infected birds can silently carry and shed the bacteria, contaminating their meat and eggs. These asymptomatic carriers are particularly problematic because they are difficult to detect, yet they play a significant role in spreading the pathogen within flocks and into human food chains. The persistent intestinal colonization by *S. enterica* in poultry is a key factor driving its transmission (2). Vaccinating chickens, along with other control strategies, is a key approach to reducing *Salmonella* prevalence in poultry flocks, thereby lowering the risk of human infections. Despite extensive research, there is still a need for *Salmonella* vaccines that are both safer and more effective (2–6). Effective vaccines should control *Salmonella* colonization in the intestines, prevent organ invasion, and decrease fecal shedding, thereby limiting the spread of infection within flocks and contamination of their products and environment (2, 3).

One promising approach to enhance vaccine efficacy is to strategically engage and activate immune cells, particularly professional antigen-presenting cells (APCs), by directly targeting the antigen to CD40 on APCs (7–9). Initially identified for its crucial role in regulating humoral immune responses, CD40 is now widely recognized as a central regulator of both cellular and humoral immunity (10). It is a costimulatory, integral glycoprotein that is mostly on APC such as dendritic cells and macrophages, and expressed throughout all stages of B cell development and differentiation, playing a crucial role in immune function (11–14). CD40L, the primary ligand for CD40 (also known as CD154, gp39, T-BAM, or TRAP), is a type II transmembrane protein primarily expressed on activated CD4+ T cells and platelets, underscoring its role in immune regulation and intercellular communication. Beyond the immune system, CD40 is also expressed in endothelial cells, fibroblasts, hematopoietic progenitors, platelets, and basal epithelial cells, demonstrating the broad physiological impact of CD40 signaling (10, 15–17). Given its central role in immune activation, CD40 has emerged as an attractive target for vaccine development, as it facilitates rapid engagement and activation of APCs, leading to a more robust and sustained immune response (11, 18–20). Targeting CD40 in vaccine design holds promise for improving T cell and B cell responses, stimulating dendritic cells for enhanced antigen presentation, increasing the pro-inflammatory activity of macrophages, promoting B cell maturation, inducing isotype switching to more effective antibodies (IgG and IgA), and promoting cytokine release to amplify the immune response.

Previous studies using a monoclonal antibody (2C5) targeting chicken CD40 have demonstrated enhanced immune activation in chickens against various antigens (8, 14, 18, 20), but their high production costs and complexity limit their widespread application, particularly in veterinary and agricultural settings. On the other hand, DNA aptamers, with their high specificity, stability, and cost-effective production, offer an attractive alternative for targeting CD40 in vaccine development. In addition, rolling Circle Amplification (RCA), an isothermal enzymatic process, has emerged as a powerful tool for producing long single-stranded DNA molecules with tandemly repeated sequences (21, 22). RCA has been effectively used to construct poly-aptamer-drug systems for targeted drug delivery and probe labeling, demonstrating its simplicity, versatility, efficiency, and applicability across various biomedical applications (23–26). Given its potential to form aptamer-based complexes with multiple target-binding units, RCA is a promising method for enhancing the efficacy of vaccine platforms.

Our previous study investigated a novel vaccine adjuvant strategy leveraging RCA products displaying anti-CD40 DNA aptamers. We conjugated the M2e epitope peptide from the influenza virus as a model hapten to the most promising Aptamer RCA (Aptamer RCA II), and evaluated the conjugate in a chicken challenge trial. The results showed that Aptamer RCA II significantly enhanced anti-M2e IgG antibody production as compared to the M2e epitope alone (11). Building on these findings, our current study focused on developing a CD40-targeting vaccine complex in which anti-*Salmonella* DNA aptamers were employed to present formalin-killed *Salmonella* as an antigen. In this study, we performed an initial evaluation of two vaccine complex designs in the HD11 macrophage cell line to identify the final design suitable for evaluation in a chicken vaccination trial.

## Materials and methods

### Selection of *Salmonella* aptamers

For antigen presentation in this vaccine design, six candidate aptamers with a reported affinity for *Salmonella* were selected from the literature: 33 (27), S8-7 (28), C4 (29), St2p (30), St1 (31), and Sal26 (32). The sequences of these aptamers are shown in Table 1. Each aptamer was synthesized with a fluorescein amidite (FAM) fluorescence label at the 5′ end (Integrated DNA Technologies, IDT, Coralville, Iowa, USA). To quantitatively assess the affinity and binding specificity of the aptamers, 50 μL of each 100 μM FAM-labeled aptamer was mixed with 450 μL of overnight cultures (10^9^ CFU/mL) of three *Salmonella* strains commonly associated with poultry: SE, *Salmonella* Typhimurium (ST), and *Salmonella* Kentucky (SK), resulting in a final aptamer concentration of 10 μM in each reaction. The *Salmonella* strains used were obtained from John Kirkpatrick Skeeles Poultry Health Laboratory (PHL) at the University of Arkansas System Division of Agriculture. The mixtures were incubated at room temperature for 1 h. with gentle shaking at 120 rpm using a Labnet Rocker 25. After 1 h of incubation, the bacterial culture was centrifuged (5,000 g) for 2 min, the supernatant was discarded, and the pellet was resuspended in sterile phosphate-buffered saline (PBS). The centrifugation and resuspension process was repeated 3 times to wash away any unbound aptamers thoroughly. Following the final wash, the aptamer-*Salmonella* complexes were resuspended in 1,000 μL of PBS. A 100 μL aliquot of each aptamer-*Salmonella* suspension was transferred to a black 96-well plate in triplicate, and fluorescence emission using an OD of 450/500 was measured in a dark room using a plate reader (Biotek synergy, Winooski, Vermont, USA). This assay was repeated 4 times, and the resulting data were analyzed to identify the top 3 aptamers with the highest binding affinity across all 3 serovars.

**Table 1:** Oligonucleotides that are used in this study.

Category	Description	DNA Sequence (5′→3′)	Number of bases	Reference
Aptamer RCA II	SEQ3	CCGAATTCGAAGGACAAGAGGTGGAATTGGTAATGGGGTGTAAATGGAGCAGTGAATTCGTCTTTTATGCTACGTCCCGC	80	(11)
	SEQ4	CCGAATTCGAAGGACAAGAGTAGGGCTACATGGAATAGGGATCAGAAGAGCAGGGCTAGGTCTTTTATGCTACGTCCCGC	80	(11)
	Template	^a^phos-GCATCTGAACGCGGGACGTAGCATAAAAGACGAATTCACTGCTCCATTTACACCCCATTACCAATTCCACCTCTTGTCCTTCGAATTCGGGATCCACCGGTAGCAGCGGGACGTAGCATAAAAGACCTAGCCCTGCTCTTCTGATCCCTATTCCATGTAGCCCTACTCTTGTCCTTCGAATTCGGGGAACGTCTT	195	(11)
	Primer	^b^bio-GTTCAGATGCAAGACGTTCC	20	(11)
	GC primer	^b^bio-GCAGGGACGAGGCGACCAGCGGGCCAG CGGGTTCAGATGCAAGACGTTCC	50	Current study
Anti-*Salmonella* RCA	33	FAM-TATGGCGGCGTCACCCGACGGGGACTTGACATTATGACAG	40	(27)
	S8-7	FAM-CTGATGTGTGGGTAGGTGTCGTTGATTTCTTCTGGTGGGG	40	(28)
	C4	FAM-ACGGGCGTGGGGGCAATGCCTGCTTGTAGGCTTCCCCTGTGCGCG	45	(29)
	St2p	FAM-ATAGGAGTCACGACGACCAGAAAGTAATGCCCGGTAGTTATTCAAAGATGAGTAGGAAAAGATATGTGCGTCTACCTCTTGACTAAT	87	(30)
	St1	FAM-CCGATGTCCGTTAGGGCTCCTCCATAGAT	29	(31)
	Sal26	FAM-TAGCTCACTCATTAGGCACATTTGTGGCACCAAATTTGAATTAATCAAGACAGTGTGGTGCATAGTTAAGCCAGCC	76	(32)
	Template	phos-CGTACCGATGCTGTCATAATGTCAAGTCCCCGTCGGGTGACGCCGCCATAGATCCACCGGTAGCACCCCACCAGAAGAAATCAACGACACCTACCCACACATCAGGATCCACCGGTAGCACGCGCACAGGGGAAGCCTACAAGCAGGCATTGCCCCCACGCCCGTCCGTAGTCAG	175	Current study
	Primer	^b^bio-CCGCTGGCCCGCTGGTCGCCTCGTCCCTGCCATCGGTACGCTGACTACGG	50	Current study
Spacer complement	Linear	GATCCACCGGTAGCA	15	(11)
	Modified 1 (33)	TATGGCGGCGTCACCCGACGGGGACTTGACATTATGACAGCTAGGTGGCCATCGT	55	Current study
	Modified 2 (S8-7)	CTGATGTGTGGGTAGGTGTCGTTGATTTCTTCTGGTGGGGCTAGGTGGCCATCGT	55	Current study
	Modified 3 (C4)	ACGGGCGTGGGGGCAATGCCTGCTTGTAGGCTTCCCCTGTGCGCGCTAGGTGGCCATCGT	60	Current study

a phos—implies templates were phosphorylated at the 5′ end.

b bio—implies primers were biotinylated at the 5′ end; GC-rich region is underlined.

Single underlines represent the primer-binding site, the GC-rich region, and the *Salmonella* aptamer in the modified spacer. Double underlines represent the spacer region.

### RCA template and primer design

**Figure 1:**
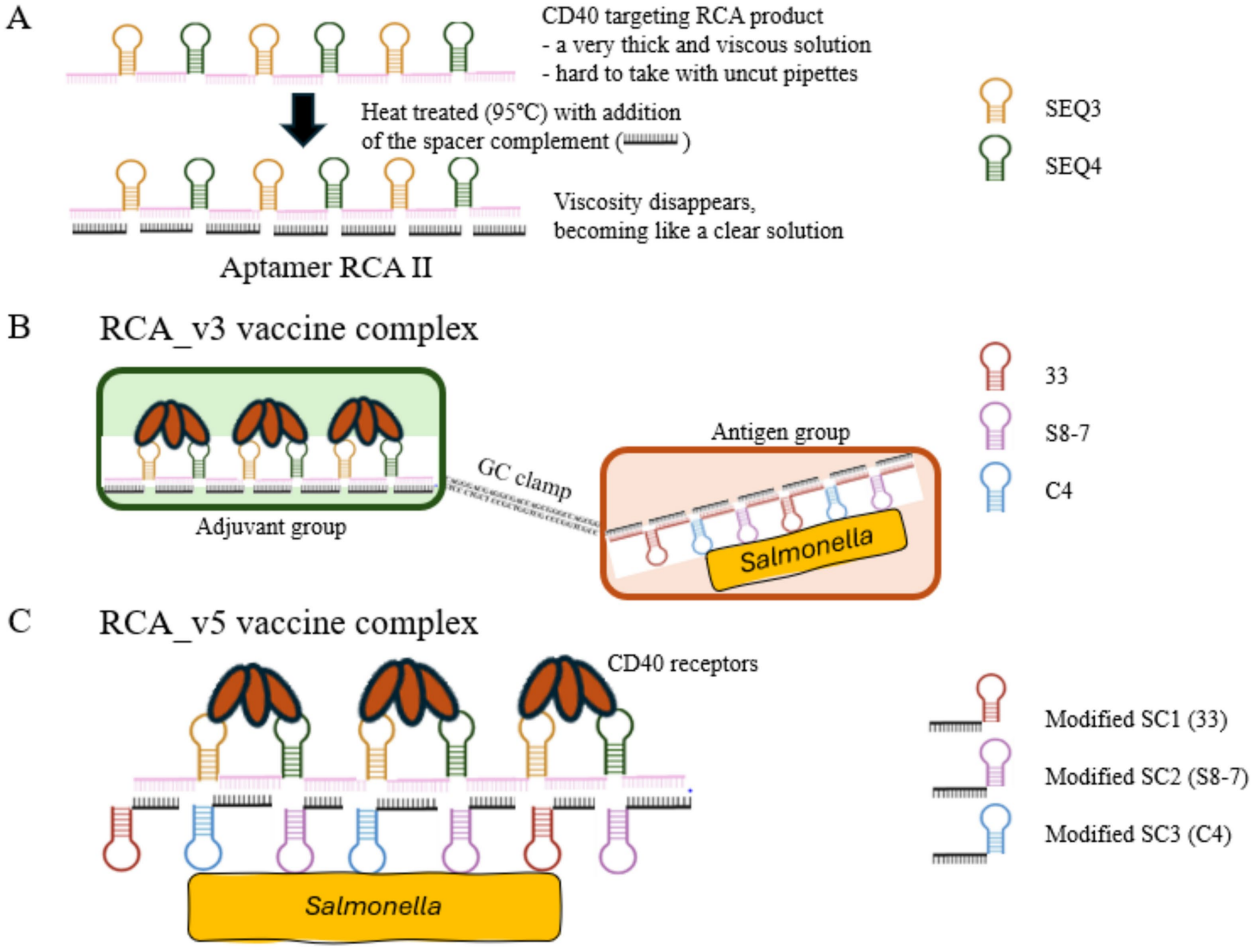
Design and assembly of RCA-based vaccine constructs. **(A)** RCA product (presenting SEQ3 and SEQ4) with spacer complement (SC) was heat-treated at 95 °C for 10 min and cooled to room temperature to form Aptamer RCA II. This procedure reduces the viscosity of the RCA product. **(B)** Schematic of the RCA_v3 vaccine construct. This design depicts the hybridization between two RCA products (Aptamer RCA II and Anti-*Salmonella* RCA) via a GC-rich clamp. Aptamer RCA II targets CD40, and anti-*Salmonella* is used to capture killed *Salmonella*. **(C)** Design of the RCA_v5 construct, which is equivalent to Aptamer RCA II except modified SCs carrying a *Salmonella*-specific aptamer in each SC at its 5′ end were used in place of normal SC, enabling targeted capture of killed *Salmonella* cells.

For the selected *Salmonella* aptamers (33, S8-7, and C4), which exhibited the highest affinity across multiple *Salmonella* serovars (see Results and discussion section), the DNA sequences of the aptamers were converted to reverse-complement and linked with spacer sequences to create an oligonucleotide template for Anti-*Salmonella* RCA. Additional sequences were incorporated at the 5′ and 3′ ends to serve as primer-binding regions for the circularization of the template and the RCA reaction. Both Aptamer RCA II and Anti-*Salmonella* RCA templates were synthesized with a phosphate group at the 5′ end to facilitate ligation, thereby producing circular templates for the RCA reaction. The DNA sequences of the RCA templates are shown in Table 1. For the primers for both RCA reactions, we designed primers with and without GC-rich sequences (50 nt) added to the 5′ end of each primer. This GC clamp was used to link the Aptamer RCA II and Anti-*Salmonella* RCA products through stable hybridization, as demonstrated in Figure 1B. All primers and templates, synthesized by Integrated DNA Technologies (IDT, Coralville, Iowa, USA), are detailed in Table 1.

Two spacer complement sequences were designed to develop two distinct vaccine constructs. The first construct used a linear spacer complement that matched the spacer regions of both Aptamer RCA II and Anti-*Salmonella* RCA, enabling efficient hybridization with the spacer regions of each RCA product (Figure 1B). In the second construct, three distinct spacer complements were created, each carrying one of the three selected *Salmonella* aptamers at the 5′ end. These modified spacers were combined in equal proportions and added to the Aptamer RCA II (Figure 1C). Unlike the first construct, the primer design for the second construct did not include a GC clamp, as the Anti-*Salmonella* RCA was directly integrated into the spacer complement. This approach was intended to facilitate more targeted interactions with *Salmonella* antigens, potentially enhancing the specificity, stability, and efficacy of the resulting vaccine complex.

### Production of rolling circle amplification (RCA) products

To produce the RCA product, the RCA primer was annealed to the template by mixing 2.5 μL of 10 μM primer and template each with 45 μL of TE buffer (pH 8.0), resulting in a final concentration of 0.5 μM in a 50 μL reaction solution (we compared the two primer concentrations, 0.5 vs. 5 μM, for RCA products, which was then tested in HD11 assay. Since there was no significant difference, we used 0.5 μM consistently throughout the study. See Supplementary Table S1.) This mixture was heated to 95 °C for 10 min, then gradually cooled to 56 °C for 5 min to promote primer-template binding. Ligation was performed with Hi-T4 DNA ligase (New England Biolabs, Ipswich, MA, USA) according to the manufacturer’s instructions, yielding a circular template. The RCA reaction was performed in a final volume of 60 μL containing the ligated template–primer complex, 2 μL of phi29 DNA polymerase (10 U/μL; New England Biolabs, Ipswich, MA, USA), 8 μL of 10× phi29 DNA polymerase buffer (New England Biolabs), 0.8 μL of 100 × bovine serum albumin (BSA; New England Biolabs), and 10 μL of 10 mM dNTPs (New England Biolabs). The mixture was incubated at 30 °C for 16 h., followed by heat inactivation at 65 °C for 10 min. phi29 DNA polymerase was used due to its high processivity and strand displacement activity, which are crucial for efficient RCA amplification (23, 33–35). RCA products were quantified using a Qubit fluorometer (Thermo Fisher Scientific, Waltham, MA, USA). Hybridization was performed by adding 5 μL of a 10 μM linear spacer complement to 50 μg of RCA product, followed by heating at 95 °C for 10 min and gradual cooling to facilitate stable complex formation. Verification of RCA amplification was performed using 1% agarose gel electrophoresis.

### Assembly of the vaccine complex

The first vaccine complex was assembled by combining 50 μg of Aptamer RCA II with 50 μg of Anti-*Salmonella* RCA and 10 μL of the spacer complement. The mixture was heated at 95 °C for 10 min and gradually cooled to room temperature to promote hybridization of the complementary GC clamps, which had been incorporated during primer design to ensure proper construct assembly.

In the second vaccine design, three spacer complements containing each of the three *Salmonella* aptamers were mixed in equal proportions, and 5 μL of this combined spacer mixture was added to 50 μg of Aptamer RCA II. The reaction was similarly heat-treated at 95 °C for 10 min and gradually cooled to facilitate binding. The viscosity of the RCA products was reduced by heat treatment at 95 °C for 10 min after spacer addition, as shown in Figure 1A. In the final step, formalin-inactivated SE cells were attached to the *Salmonella* RCA component via aptamer binding to generate the complete vaccine complex. The resulting RCA vaccine complexes were designated RCA_v3 (GC clamp) and RCA_v5 (modified space complements with attached anti-*Salmonella* aptamers), respectively, as illustrated in Figure 1.

### Quantification of *Salmonella* binding to RCA vaccine construct

To quantify the number of SE cells that bind to anti-*Salmonella* RCA products in both vaccine constructs, we used DNase I digestion to release the captured target cells as previously described (36). An overnight culture of SE was prepared, washed three times, and resuspended in 1,000 μL of sterile PBS. Four experimental groups were prepared and labeled: RCA_v3 without DNase I, RCA_v3 with DNase I, RCA_v5 without DNase I, and RCA_v5 with DNase I. All RCA constructs were synthesized using primers modified with biotin at the 5′ end to enable binding to streptavidin-coated magnetic beads. Each tube containing the designated vaccine construct received 100 μL of the SE suspension and was incubated at room temperature for 1 h. with gentle shaking at 120 rpm using a Labnet Rocker 25. In parallel, a 10-fold dilution of the SE culture was plated to determine the starting bacterial concentration. After the initial incubation, 200 μL of pre-equilibrated streptavidin-coated magnetic beads (Thermo Fisher Scientific, Waltham, MA, USA) were added to each tube, followed by a 1 h incubation at room temperature with gentle shaking. Tubes were then washed five times with sterile PBS using a magnetic rack to remove unbound bacteria. DNase I treatment was performed by resuspending the washed pellet in 80 μL sterile water, 10 μL 10 × DNase I buffer, and 10 μL DNase I (New England Biolabs) to facilitate DNA digestion and release of bound *Salmonella*. Control tubes without DNase I received 90 μL of sterile water and 10 μL of 10 × buffer. All tubes were incubated at 37 °C for 25 min. Following incubation, the supernatant was removed using a magnetic rack, and 10-fold serial dilutions were prepared; 50 μL from each dilution was plated on LB agar. Plates were incubated at 37 °C for 24 h, and colony counts were used to estimate the number of bacteria bound to each RCA construct.

### HD11 assay to quantify macrophage activation by the RCA vaccine complexes

The HD11 chicken macrophage cell line was obtained from Dr. Luc R. Berghman’s lab at Texas A&M University. The cells were cultured using a T-75 tissue culture flask (VWR, Radnor, PA, USA) in Dulbecco’s Modification of Eagle’s Medium (DMEM, Thermo Fisher Scientific, Waltham, MA, USA) supplemented with 8% fetal bovine serum (FBS, Thermo Fisher Scientific), 5% chicken serum (Thermo Fisher Scientific), 1% GlutaMAX (Thermo Fisher Scientific), and 1% penicillin–streptomycin (Thermo Fisher Scientific).

HD11 chicken macrophage cells (passage 7) were cultured at 37 °C in a humidified atmosphere containing 5% CO₂. Cells were seeded at a density of 1 × 10^6^ cells per well in a 24-well tissue culture plate and allowed to adhere for 8 h. After adherence, the culture medium was replaced with 1 mL of the appropriate treatment, diluted in growth medium. Negative controls included growth medium alone and pre-immune mouse serum (1:300; Jackson ImmunoResearch, West Grove, PA, USA), while lipopolysaccharide (LPS; 5 μg/mL, Millipore Sigma, St. Louis, MO, USA) served as a positive control for macrophage activation. Additional treatment groups included Aptamer RCA II, RCA_v3, SE alone, RCA_v3 vaccine complex, and RCA_v5 vaccine complex. Each treatment was performed in 4 independent biological replicates (4 separate independent experiments per treatment). For each biological replicate, nitric oxide levels were quantified using 2 technical replicates. The plates were incubated for 18 h, centrifuged at 500 × *g* for 10 min, and the supernatant was collected for nitric oxide (NO) analysis. Nitric oxide levels in the supernatant were determined using the Griess assay as described by Green et al. (37) and Crippen et al. (38). A sodium nitrite standard curve (0–80 μM) was prepared by serial dilution of a 2 mM stock solution in unsupplemented growth medium. Standards and supernatant samples (100 μL each) were added in triplicate to a 96-well flat-bottom plate, followed by 50 μL of Reagent 1 (1% sulfanilamide in 2.5% phosphoric acid). Plates were incubated for 10 min at room temperature in the dark, after which 50 μL of Reagent 2 (0.1% naphthylenediamine in 2.5% phosphoric acid) was added and incubated for another 10 min. Absorbance was measured at 540 nm using a microplate reader (Biotek Synergy, Winooski, VT, USA), and NO concentrations were calculated from the standard curve.

### Statistical analysis

All data were analyzed using one-way ANOVA in JMP Pro 18. Means were further separated using Tukey–Kramer HSD, and significance was set at *p* ≤ 0.05.

## Results and discussion

### Rationale for the design of the RCA vaccine complexes

In our previous study, we employed the streptavidin-biotin interaction to conjugate Aptamer RCA II with the M2e peptide, aiming to enhance immune responses against the viral pathogen in chickens. Avidin is a tetrameric glycoprotein originally isolated from egg white, and it exhibits exceptionally high affinity and specificity for biotin. This property underlies the avidin-biotin system, which has been widely adopted in molecular biology and vaccine development for conjugating functional components (39–41).

Although effective for molecular assembly, the avidin-biotin system does not stimulate the immune system and has raised regulatory concerns. Notably, the U.S. Food and Drug Administration (FDA) has cautioned against its extensive use, as excess biotin can interfere with clinical immunoassays, leading to inaccurate diagnostic results (39).

To address these safety and regulatory concerns, we devised two alternative conjugation strategies for *Salmonella* vaccine complexes. For RCA_v3 (Figure 1B), two complementary high-GC regions were incorporated directly at the 5′ end of the RCA primer to produce two biotinylated RCA products, which can be joined by hybridization, forming a GC clamp (42–44). For RCA_v5 (Figure 1C), we engineered a complementary spacer sequence within the *Salmonella* Aptamer RCA, allowing direct hybridization with Aptamer RCA II via a spacer region. These modifications eliminate the streptavidin-biotin component, mitigating its potential adverse effects and simplifying large-scale poultry vaccination production.

### Characterization of the binding efficiency of selected *Salmonella*-specific aptamers

To incorporate whole killed *Salmonella* cells into the vaccine complex, it was essential to capture and anchor them to the CD40-targeting component. In this study, we evaluated the potential of anti-*Salmonella* DNA aptamers for this purpose. Based on a literature review, six previously reported DNA aptamers that bind various *Salmonella* serotypes were selected (Table 1). These aptamers were tested for binding affinity against three relevant serovars—SE, ST, and SK—to identify those with the strongest binding capacity against these 3 serovars.

Various methods have been employed to assess aptamer-cell binding interactions, including flow cytometry (45–47), SELEX-based assay (28, 48, 49), and confocal imaging (46). Additionally, Joshi et al. (27) employed Electrophoretic Mobility Shift Analysis (EMSA), mass spectrometry, and DNase footprinting to study aptamer binding to ST outer membrane protein (OMP). In our study, we employed a fluorescence spectrophotometry-based assay, chosen for its ease of use, rapid turnaround, high sensitivity and selectivity, and cost-effectiveness (50, 51).

The results, presented in Supplementary Figure S1, show that aptamers St2p, Sal26, and St1 demonstrated minimal binding affinity across all three *Salmonella* serovars (≤2.750), often at very low levels for ST. In contrast, aptamers S8-7, 33, and C4 exhibited stronger binding affinity across all serovars, with aptamer C4 displaying the highest binding affinity, particularly to ST (19.000), consistent with the findings by Moon et al. (29). Aptamers 33 and S8-7 demonstrated moderate binding compared to C4, with binding values ranging from 6.000 to 9.750, in agreement with the previous result (28, 72).

These findings informed the selection of aptamers S8-7, 33, and C4 for further application in vaccine design. Their ability to bind multiple *Salmonella* serovars suggests potential for broad-spectrum vaccine applications, addressing a critical need for comprehensive *Salmonella* control in the poultry industry.

### Verification of the optimized high-yield RCA products

Agarose gel electrophoresis is one of the most common methods for validating and visualizing RCA product formation (23, 52–56). Other visualization techniques include fluorescence-based methods such as microscopy, flow cytometry, and fluorescence spectroscopy, using dye-labeled probes (24, 53, 57–60), and nanoparticle-based platforms, including magnetic beads and quantum dots (61, 62).

**Figure 2:**
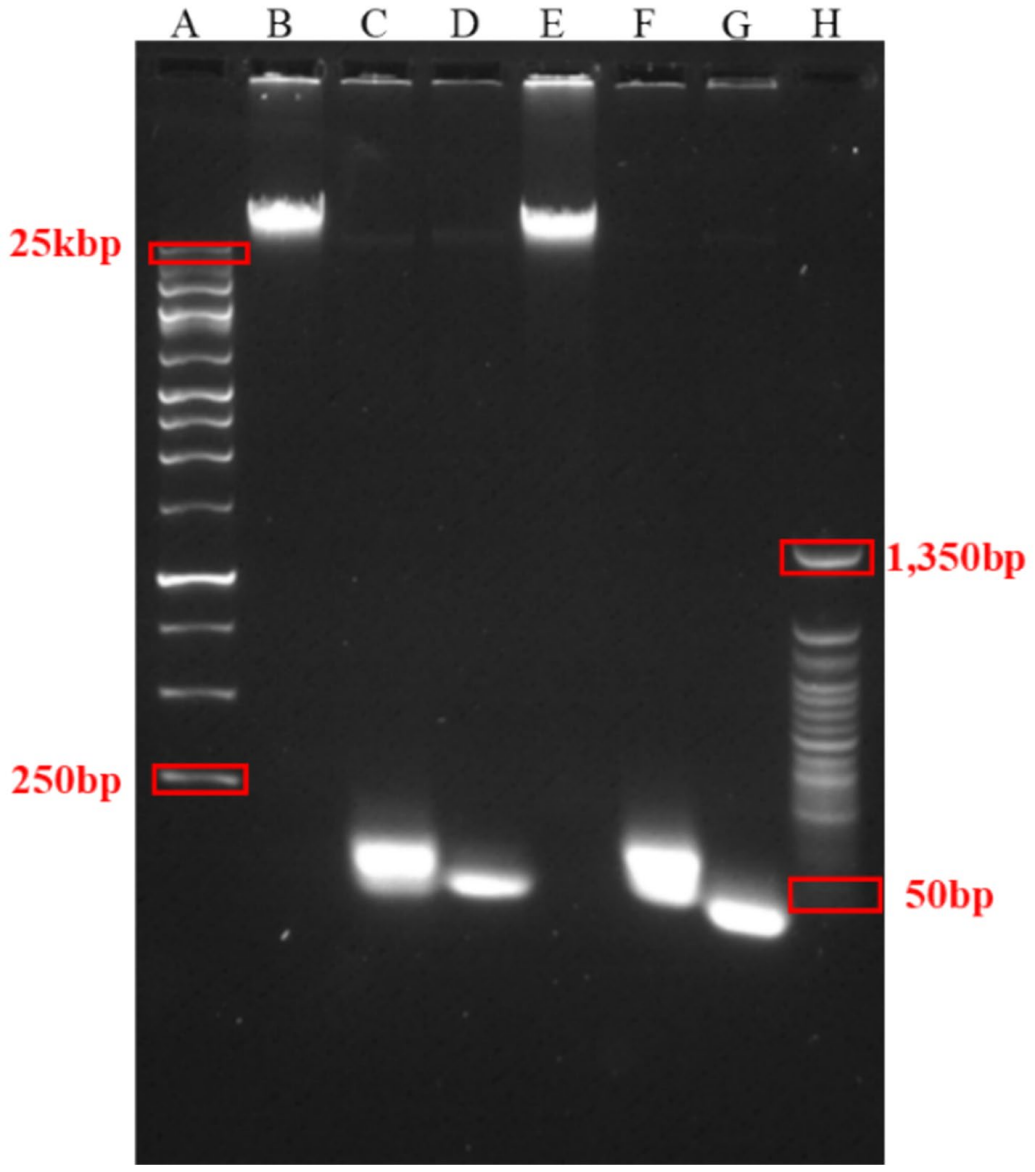
Gel electrophoresis of RCA products. RCA reactions were analyzed on a 1% TAE agarose gel. Lane A: 25 kb DNA ladder; Lane B: anti-*Salmonella* RCA (10× dilution); Lane C: anti-*Salmonella* RCA without primer; Lane D: anti-*Salmonella* RCA without template; Lane E: Aptamer RCA II (10× dilution); Lane F: Aptamer RCA II without primer; Lane G: Aptamer RCA II without template; Lane H: 50 bp DNA ladder.

In this study, RCA products were assessed on a 1.0% TAE agarose gel (Figure 2). Lane A contains a 25-kb DNA ladder as a molecular weight reference. Lanes B and E, corresponding to 10x diluted anti-*Salmonella* RCA and Aptamer RCA II, respectively, display high-molecular-weight bands (>25 kb), confirming successful amplification (52, 53, 55). Control lanes included RCA reactions performed without primers (lanes C and F) and without templates (lanes D and G) for both RCA constructs. These lanes showed only low-molecular-weight bands, indicating the absence of RCA amplification. Lane H, containing a 50 bp ladder, provided size approximation for smaller bands. The presence of distinct high-molecular-weight bands in the experimental lanes and their absence in the controls confirms the successful generation of high-yield RCA products.

### Quantification of *Salmonella* binding to RCA constructs for vaccine dose selection

To optimize the vaccine complex for SE binding, we quantified the colony-forming units (CFU) of SE bound to each vaccine construct (RCA_v3 and RCA_v5, with or without DNase I treatment). It was accomplished using streptavidin magnetic beads to capture biotinylated RCA products containing bound SE. The strong, specific interaction between biotin and streptavidin made streptavidin-coated magnetic beads an effective tool for isolating biotin-labeled DNA–probe hybrids (63). The initial SE dose used for binding was 4.6 × 10^8^ CFU. For RCA_v3, DNase I treatment markedly increased bacterial binding from 9.6 ± 0.5 × 10^5^ CFU in the untreated sample to 3.1 ± 0.2 × 10^6^ CFU, representing an approximate increase of 2.1 × 10^6^ CFU. Similarly, RCA_v5 exhibited enhanced binding following DNase I treatment, increasing from 4.3 ± 0.4 × 10^5^ CFU in the untreated group to 3.2 ± 0.3 × 10^6^ CFU, a difference of about 2.8 × 10^6^ CFU (Supplementary Figure S2). These results represent the mean ± standard deviation of 4 independent experiments conducted on separate days using independently prepared samples, without additional technical replication (*p* < 0.05). Based on these binding results, and consistent with previous studies reporting that *Salmonella* doses within the range of 10^5^–10^8^ CFU induce robust immune responses in poultry, a dose of 1 × 10^7^ CFU was selected for vaccination (64–66). However, future studies may incorporate fluorescent labeling and flow cytometry–based approaches to further characterize RCA–bacteria binding dynamics in addition to CFU-based viability quantification.

### Macrophage activation by RCA-based vaccine complexes

**Figure 3:**
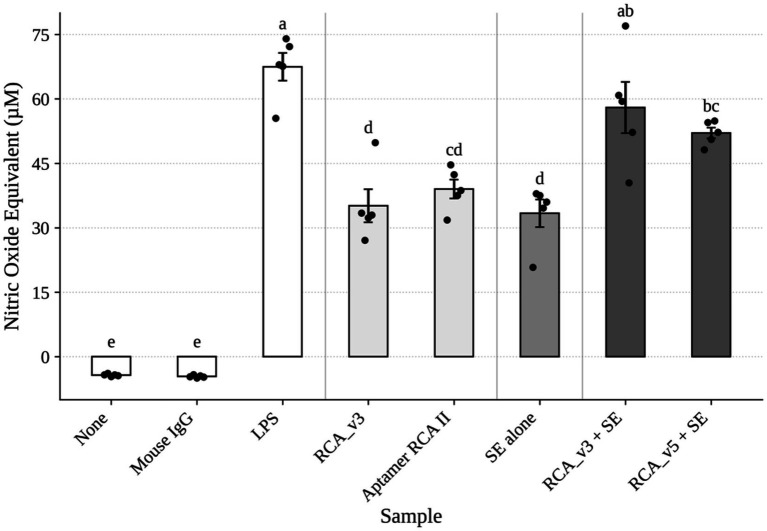
Nitric oxide assay using the HD11 cell line to evaluate the RCA vaccine. The nitric oxide assay results were grouped into four categories. The first category consisted of controls: negative controls included cells treated with growth media (none) and mouse IgG, both of which showed no macrophage activation. LPS served as the positive control due to its well-established ability to activate macrophages. The second category included RCA complexes without killed *Salmonella enteritidis* (SE), specifically RCA_v3 and Aptamer RCA II. The third category is killed SE alone. The fourth category consisted of RCA vaccine complexes containing killed SE, RCA_v3, and RCA_v5 (RCA_v3 + SE and RCA_v5 + SE, respectively). Bars sharing the same letter are not significantly different (*p* < 0.05).

The production of nitric oxide by chicken macrophages plays a key role in the innate immune response as a defense mechanism against bacterial invasion (38). The HD11 macrophage cell line is a well-established model for assessing macrophage activation via nitric oxide production (11, 67, 68). In this study, HD11 cells were treated with vaccine formulations to evaluate their immunostimulatory potential. Medium alone and mouse IgG served as negative controls and induced minimal nitric oxide production (Figure 3). In contrast, lipopolysaccharide (LPS), used as a positive control, elicited the highest level of nitric oxide production, consistent with its known role as a potent macrophage activator (69, 70).

The RCA complex without inactivated SE (Aptamer RCA II and RCA_v3) and inactivated SE alone showed moderate levels of macrophage activation, with no significant difference between them.

However, both RCA_v3 and RCA_v5 vaccine complexes demonstrated significantly higher macrophage activation compared to the RCA complex without inactivated SE (RCA only) (*p* < 0.05). Although the difference between RCA_v3 and RCA_v5 was not statistically significant, RCA_v3 induced a numerically greater response (Figure 3). This *in vitro* activation of macrophages, evidenced by increased nitric oxide production, highlights the potential of the RCA-based vaccine complexes (RCA_v3 and RCA_v5) to engage CD40 receptors on antigen-presenting cells. The enhanced NO production following stimulation with RCA constructs incorporating inactivated SE highlights the importance of combining antigen presentation with CD40 targeting to boost immune activation, an effect that may be critical for initiating protective adaptive immunity *in vivo*.

It is important to note that in our previous study (11), we included NC RCA product, which is the same type of RCA product but using two random aptamer sequences (which were not enriched during SELEX cycles) in the HD11 assay as a negative control. Indeed, the result showed that NC RCA failed to activate HD11 cells, suggesting that the activation by Aptamer RCA II, RCA_v3, and RCA_v5 shown in this study (Figure 3) is due to the specific aptamers (SEQ3 and SEQ4) present in these RCA products. Although we cannot exclude the possibility of HD11 activation through other pathways or receptors (without further experimental data), the specificity of SEQ3 and SEQ4 for chCD40 and their requirement for activating HD11 strongly suggest that CD40 receptors are the major pathway of HD11 activation in the current study.

## Conclusion

In this study, DNA-based Aptamer RCA vaccine complexes targeting CD40 were developed and evaluated for their potential to control *Salmonella* colonization in chickens. The results demonstrated their ability to activate macrophage cells *in vitro* (Figure 3). The results highlight the vaccine’s potential as an effective strategy for controlling *Salmonella* colonization in broiler chickens, which is a critical issue in poultry production and food safety. By combining the specificity and stability of DNA aptamers with the scalability of the high-yield RCA process, this novel vaccine design may provide a promising solution to control *Salmonella* in the poultry industry.

While both RCA_v3 and RCA_v5 vaccine complex designs showed promising results *in vitro*, a future study evaluating vaccine efficacy in a chicken vaccination study with *Salmonella* challenge is necessary to demonstrate their efficacy in chickens. Future research could also include measuring immune responses, such as IgM, IgY, and IgA responses, and cell uptake analyses (including macrophage and dendritic cell binding/uptake assays). Overall, this study highlights a novel, easy-to-make vaccination strategy for controlling *Salmonella* in poultry, with potential applications in veterinary and agricultural settings.

## Data Availability

The original contributions presented in the study are included in the article/Supplementary material, further inquiries can be directed to the corresponding author.

## References

[1] Shaji S Selvaraj RK Shanmugasundaram R. *Salmonella* infection in poultry: a review on the pathogen and control strategies. Microorganisms. (2023) 11:2814. doi: 10.3390/microorganisms11112814, 38004824 PMC10672927

[2] Kogut MH Santin E. "Advances in vaccines for controlling foodborne *Salmonella* spp. in poultry". In: K Venkitanarayanan S Thakur S Ricke, editors. Food Safety in Poultry Meat Production. Cham, Switzerland: Spring Nature. (2019). p. 161–89.

[3] Desin TS Köster W Potter AA. Salmonella vaccines in poultry: past, present and future. Expert Rev Vaccines. (2013) 12:87–96. doi: 10.1586/erv.12.138 23256741

[4] Jajere SM. A review of *Salmonella enterica* with particular focus on the pathogenicity and virulence factors, host specificity and antimicrobial resistance including multidrug resistance. Vet World. (2019) 12:504–21. doi: 10.14202/vetworld.2019.504-521, 31190705 PMC6515828

[5] Sarrami Z Sedghi M Mohammadi I Bedford M Miranzadeh H Ghasemi R. Effects of bacteriophage on *Salmonella* Enteritidis infection in broilers. Sci Rep. (2023) 13:12198. doi: 10.1038/s41598-023-38791-6, 37500690 PMC10374914

[6] Van Immerseel F Methner U Rychlik I Nagy B Velge P Martin G . Vaccination and early protection against non-host-specific Salmonella serotypes in poultry: exploitation of innate immunity and microbial activity. Epidemiol Infect. (2005) 133:959–78. doi: 10.1017/S0950268805004711, 16274493 PMC2870330

[7] Berry JD Licea A Popkov M Cortez X Fuller R Elia M . Rapid monoclonal antibody generation via dendritic cell targeting in vivo. Hybrid Hybridomics. (2003) 22:23–31. doi: 10.1089/153685903321538053, 12713687

[8] Chen C-H Abi-Ghanem D Waghela SD Chou W-K Farnell MB Mwangi W . Immunization of chickens with an agonistic monoclonal anti-chicken CD40 antibody–hapten complex: rapid and robust IgG response induced by a single subcutaneous injection. J Immunol Methods. (2012) 378:116–20. doi: 10.1016/j.jim.2012.02.006, 22366632

[9] Hatzifoti C Heath AW. CD40-mediated enhancement of immune responses against three forms of influenza vaccine. Immunology. (2007) 122:98–106. doi: 10.1111/j.1365-2567.2007.02617.x, 17472718 PMC2265986

[10] van Kooten C Banchereau J. CD40–CD40 ligand. J Leukoc Biol. (2000) 67:2–17. doi: 10.1002/jlb.67.1.2 10647992

[11] Al-Ogaili AS Liyanage R Lay JO Jiang T Vuong CN Agrawal S . DNA aptamer-based rolling circle amplification product as a novel immunological adjuvant. Sci Rep. (2020) 10:22282. doi: 10.1038/s41598-020-79420-w, 33335251 PMC7747709

[12] Banchereau J Bazan F Blanchard D Briere F Galizzi JP Kooten C . The CD40 antigen and its ligand. Annu Rev Immunol. (1994) 12:881–926. doi: 10.1146/annurev.iy.12.040194.004313 7516669

[13] Bullock TN. CD40 stimulation as a molecular adjuvant for cancer vaccines and other immunotherapies. Cell Mol Immunol. (2022) 19:14–22. doi: 10.1038/s41423-021-00734-4, 34282297 PMC8752810

[14] Chen C-H Abi-Ghanem D Njongmeta L Bray J Mwangi W Waghela SD . Production and characterization of agonistic monoclonal antibodies against chicken CD40. Devel Comp Immunol. (2010) 34:1139–43. doi: 10.1016/j.dci.2010.06.014, 20599554

[15] Armitage RJ Fanslow WC Strockbine L Sato TA Clifford KN Macduff BM . Molecular and biological characterization of a murine ligand for CD40. Nature. (1992) 357:80–2. doi: 10.1038/357080a0 1374165

[16] Elgueta R Benson MJ De Vries VC Wasiuk A Guo Y Noelle RJ. Molecular mechanism and function of CD40/CD40L engagement in the immune system. Immunol Rev. (2009) 229:152–72. doi: 10.1111/j.1600-065X.2009.00782.x, 19426221 PMC3826168

[17] Vonderheide RH. Prospect of targeting the CD40 pathway for cancer therapy. Clin Cancer Res. (2007) 13:1083–8. doi: 10.1158/1078-0432.CCR-06-1893, 17317815

[18] Chou W-K Chen C-H Vuong CN Abi-Ghanem D Waghela SD Mwangi W . Significant mucosal sIgA production after a single oral or parenteral administration using in vivo CD40 targeting in the chicken. Res Vet Sci. (2016) 108:112–5. doi: 10.1016/j.rvsc.2016.08.013, 27663378

[19] Omolewu A. Development of Aptamer Rolling Circle Amplification Platform as a Vaccine Adjuvant to Control *Salmonella* in Broiler Chickens. Fayetteville, Arkansas, U.S.: University of Arkansas (2021).

[20] Vuong CN Chou W-K Briggs W Faulkner O Wolfenden A Jonas M . Crude inactivated influenza a virus adjuvated with a bispecific antibody complex targeting chicken CD40 and AIV M2e confers protection against lethal HPAI challenge in chickens. Monoclon Antib Immunodiagn Immunother. (2018) 37:245–51. doi: 10.1089/mab.2018.0040, 30592705

[21] Demidov VV. "Introduction: 20+ years of rolling the DNA minicircles—state of the art in the RCA-based nucleic acid diagnostics and therapeutics". In: VV Demidov, editor. Rolling Circle Amplification (RCA) Toward New Clinical Diagnostics and Therapeutics. Cham Switzerland: Springer International Publishing AG. (2016). p. 1–7.

[22] Goo N-I Kim D-E. Rolling circle amplification as isothermal gene amplification in molecular diagnostics. Biochip J. (2016) 10:262–71. doi: 10.1007/s13206-016-0402-6, 32226587 PMC7096790

[23] Ali MM Li F Zhang Z Zhang K Kang D-K Ankrum JA . Rolling circle amplification: a versatile tool for chemical biology, materials science and medicine. Chem Soc Rev. (2014) 43:3324–41. doi: 10.1039/c3cs60439j, 24643375

[24] Berr A Schubert I. Direct labelling of BAC-DNA by rolling-circle amplification. Plant J. (2006) 45:857–62. doi: 10.1111/j.1365-313X.2005.02637.x, 16460517

[25] Demidov VV. Rolling-circle amplification in DNA diagnostics: the power of simplicity. Expert Rev Mol Diagn. (2002) 2:542–8. doi: 10.1586/14737159.2.6.542, 12465451

[26] Zhang Z Ali MM Eckert MA Kang DK Chen YY Sender LS . A polyvalent aptamer system for targeted drug delivery. Biomaterials. (2013) 34:9728–35. doi: 10.1016/j.biomaterials.2013.08.079, 24044994

[27] Joshi R Janagama H Dwivedi HP Senthil Kumar TMA Jaykus L-A Schefers J . Selection, characterization, and application of DNA aptamers for the capture and detection of *Salmonella enterica* serovars. Mol Cell Probes. (2009) 23:20–8. doi: 10.1016/j.mcp.2008.10.006, 19049862

[28] Dwivedi HP Smiley RD Jaykus L-A. Selection of DNA aptamers for capture and detection of *Salmonella* Typhimurium using a whole-cell SELEX approach in conjunction with cell sorting. Appl Microbiol Biotechnol. (2013) 97:3677–86. doi: 10.1007/s00253-013-4766-4, 23494620

[29] Moon J Kim G Lee S Park S. Identification of *Salmonella Typhimurium*-specific DNA aptamers developed using whole-cell SELEX and FACS analysis. J Microbiol Methods. (2013) 95:162–6. doi: 10.1016/j.mimet.2013.08.005, 23978634

[30] Duan N Wu S Chen X Huang Y Xia Y Ma X . Selection and characterization of aptamers against *Salmonella* Typhimurium using whole-bacterium systemic evolution of ligands by exponential enrichment (SELEX). J Agric Food Chem. (2013) 61:3229–34. doi: 10.1021/jf400767d, 23473545

[31] Park H-C Baig IA Lee S-C Moon J-Y Yoon M-Y. Development of ssDNA Aptamers for the sensitive detection of Salmonella typhimurium and *Salmonella enteritidis*. Appl Biochem Biotechnol. (2014) 174:793–802. doi: 10.1007/s12010-014-1103-z, 25096391

[32] Lavu PSR Mondal B Ramlal S Murali HS Batra HV. Selection and characterization of aptamers using a modified whole cell bacterium SELEX for the detection of *Salmonella enterica* Serovar Typhimurium. ACS Comb Sci. (2016) 18:292–301. doi: 10.1021/acscombsci.5b00123, 27070414

[33] Berman AJ Kamtekar S Goodman JL Lazaro JM de Vega M Blanco L . Structures of phi29 DNA polymerase complexed with substrate: the mechanism of translocation in B-family polymerases. EMBO J. (2007) 26:3494–505. doi: 10.1038/sj.emboj.7601780, 17611604 PMC1933411

[34] Lin C Xie M Chen J Liu Y Yan H. Rolling-circle amplification of a DNA nanojunction. Angew Chem Int Ed. (2006) 45:7537–9. doi: 10.1002/anie.200602113, 17048296

[35] Rodríguez I Lázaro JM Blanco L Kamtekar S Berman AJ Wang J . A specific subdomain in φ29 DNA polymerase confers both processivity and strand-displacement capacity. Proc Natl Acad Sci. (2005) 102:6407–12. doi: 10.1073/pnas.0500597102, 15845765 PMC1088371

[36] Zhao W Cui CH Bose S Guo D Shen C Wong WP . Bioinspired multivalent DNA network for capture and release of cells. Proc Natl Acad Sci USA. (2012) 109:19626–31. doi: 10.1073/pnas.1211234109, 23150586 PMC3511714

[37] Green LC Wagner DA Glogowski J Skipper PL Wishnok JS Tannenbaum SR. Analysis of nitrate, nitrite, and [15N] nitrate in biological fluids. Anal Biochem. (1982) 126:131–8. doi: 10.1016/0003-2697(82)90118-X 7181105

[38] Crippen TL Sheffield CL He H Lowry VK Kogut MH. Differential nitric oxide production by chicken immune cells. Dev Comp Immunol. (2003) 27:603–10. doi: 10.1016/S0145-305X(03)00035-1 12697316

[39] Balzer AH Whitehurst CB. An analysis of the biotin–(strept) avidin system in immunoassays: interference and mitigation strategies. Curr Issues Mol Biol. (2023) 45:8733–54. doi: 10.3390/cimb45110549, 37998726 PMC10670868

[40] Lu Z Zhang Y Wang Y Tan G-H Huang F-Y Cao R . A biotin-avidin-system-based virus-mimicking nanovaccine for tumor immunotherapy. J Control Release. (2021) 332:245–59. doi: 10.1016/j.jconrel.2021.02.029, 33647430

[41] Purow B Staveley-O'Carroll K. Targeting of vaccinia virus using biotin-avidin viral coating and biotinylated antibodies. J Surg Res. (2005) 123:49–54. doi: 10.1016/j.jss.2004.04.022, 15652950

[42] Lin K-Y Matteucci MD. A cytosine analogue capable of clamp-like binding to a guanine in helical nucleic acids. J Am Chem Soc. (1998) 120:8531–2. doi: 10.1021/ja981286z

[43] Lou C Dallmann A Marafini P Gao R Brown T. Enhanced H-bonding and π-stacking in DNA: a potent duplex-stabilizing and mismatch sensing nucleobase analogue. Chem Sci. (2014) 5:3836–44. doi: 10.1039/C4SC00948G

[44] Wilds CJ Maier MA Tereshko V Manoharan M Egli M. Direct observation of a cytosine analogue that forms five hydrogen bonds to guanosine: guanidino G-clamp. Angew Chem Int Ed. (2002) 41:115–7. doi: 10.1002/1521-3773(20020104)41:1<115::AID-ANIE115>3.0.CO;2-R, 12491456

[45] Lee Y Han S Maeng J Cho Y Lee S. In vitro selection of *Escherichia coli* O157:H7-specific RNA aptamer. Biochem Biophys Res Commun. (2012) 417:414–20. doi: 10.1016/j.bbrc.2011.11.130, 22166202

[46] Shangguan D Li Y Tang Z Cao ZC Chen HW Mallikaratchy P . Aptamers evolved from live cells as effective molecular probes for cancer study. Proc Natl Acad Sci. (2006) 103:11838–43. doi: 10.1073/pnas.0602615103, 16873550 PMC1567664

[47] Wang K Zeng Y Yang X Li W Lan X. Utility of aptamer-fluorescence in situ hybridization for rapid detection of *Pseudomonas aeruginosa*. Eur J Clin Microbiol Infect Dis. (2011) 30:273–8. doi: 10.1007/s10096-010-1074-0, 20936492

[48] Dwivedi H Smiley R Jaykus L. Selection and characterization of DNA aptamers with binding selectivity to *Campylobacter jejuni* using whole-cell SELEX. Appl Microbiol Biotechnol. (2010) 87:2323–34. doi: 10.1007/s00253-010-2728-7, 20582587

[49] Liu G Yu X Xue F Chen W Ye Y Yang X . Screening and preliminary application of a DNA aptamer for rapid detection of *Salmonella* O8. Microchim Acta. (2012) 178:237–44. doi: 10.1007/s00604-012-0825-2

[50] Lee M Shin S Kim S Park N. Recent advances in biological applications of aptamer-based fluorescent biosensors. Molecules. (2023) 28:7327. doi: 10.3390/molecules28217327, 37959747 PMC10647268

[51] Weaver SD Whelan RJ. Characterization of DNA aptamer–protein binding using fluorescence anisotropy assays in low-volume, high-efficiency plates. Anal Methods. (2021) 13:1302–7. doi: 10.1039/D0AY02256J, 33533761

[52] Ali MM Li Y. Colorimetric sensing by using allosteric-DNAzyme-coupled rolling circle amplification and a peptide nucleic acid–organic dye probe. Angew Chem. (2009) 121:3564–7. doi: 10.1002/ange.200805966 19360817

[53] Ali MM Su S Filipe CD Pelton R Li Y. Enzymatic manipulations of DNA oligonucleotides on microgel: towards development of DNA–microgel bioassays. Chem Commun. (2007) 43:4459–61. doi: 10.1039/b709817k 17971955

[54] Qi X Bakht S Devos KM Gale MD Osbourn A. L-RCA (ligation-rolling circle amplification): a general method for genotyping of single nucleotide polymorphisms (SNPs). Nucleic Acids Res. (2001) 29:e116. doi: 10.1093/nar/29.22.e116, 11713336 PMC92587

[55] Zhang T Qing T Xiao-Jun B Qian C Juan Y. Rapid visualized detection of *Escherichia coli* O157: H7 by DNA hydrogel based on rolling circle amplification. Chin J Anal Chem. (2021) 49:377–86. doi: 10.1016/S1872-2040(21)60085-3

[56] Zhao W Gao Y Kandadai SA Brook MA Li Y. DNA polymerization on gold nanoparticles through rolling circle amplification: towards novel scaffolds for three-dimensional periodic nanoassemblies. Angew Chem. (2006) 118:2469–73. doi: 10.1002/ange.200600061 16526071

[57] Cho EJ Yang L Levy M Ellington AD. Using a deoxyribozyme ligase and rolling circle amplification to detect a non-nucleic acid analyte, ATP. J Am Chem Soc. (2005) 127:2022–3. doi: 10.1021/ja043490u, 15713061

[58] Göransson J Ke R Nong RY Howell WM Karman A Grawe J . Rapid identification of bio-molecules applied for detection of biosecurity agents using rolling circle amplification. PLoS One. (2012) 7:e31068. doi: 10.1371/journal.pone.0031068, 22383994 PMC3285169

[59] Linck L Reiß E Bier F Resch-Genger U. Direct labeling rolling circle amplification as a straightforward signal amplification technique for biodetection formats. Anal Methods. (2012) 4:1215–20. doi: 10.1039/c2ay05760c

[60] Sato K Ishii R Sasaki N Sato K Nilsson M. Bead-based padlock rolling circle amplification for single DNA molecule counting. Anal Biochem. (2013) 437:43–5. doi: 10.1016/j.ab.2013.02.016, 23467098

[61] Ali MM Kanda P Aguirre SD Li Y. Modulation of DNA-modified gold-nanoparticle stability in salt with concatemeric single-stranded DNAs for colorimetric bioassay development. Chem Eur J. (2011) 17:2052–6. doi: 10.1002/chem.201002677 21294175

[62] Xu W Xie X Li D Yang Z Li T Liu X. Ultrasensitive colorimetric DNA detection using a combination of rolling circle amplification and nicking endonuclease-assisted nanoparticle amplification (NEANA). Small. (2012) 8:1846–50. doi: 10.1002/smll.201200263, 22461378

[63] St John J Quinn TW. Rapid capture of DNA targets. BioTechniques. (2008) 44:259–64. doi: 10.2144/000112633 18330355

[64] Barrow PA Huggins MB Lovell MA. Host specificity of Salmonella infection in chickens and mice is expressed in vivo primarily at the level of the reticuloendothelial system. Infect Immun. (1994) 62:4602–10. doi: 10.1128/iai.62.10.4602-4610.1994, 7927727 PMC303149

[65] Gantois I Ducatelle R Pasmans F Haesebrouck F Gast R Humphrey TJ . Mechanisms of egg contamination by *Salmonella* Enteritidis. FEMS Microbiol Rev. (2009) 33:718–38. doi: 10.1111/j.1574-6976.2008.00161.x 19207743

[66] Hassan JO Curtiss R. Development and evaluation of an experimental vaccination program using a live avirulent *Salmonella typhimurium* strain to protect immunized chickens against challenge with homologous and heterologous Salmonella serotypes. Infect Immun. (1994) 62:5519–27. doi: 10.1128/iai.62.12.5519-5527.1994, 7960134 PMC303297

[67] Lin AW Chang CC McCormick CC. Molecular cloning and expression of an avian macrophage nitric-oxide synthase cDNA and the analysis of the genomic 5′-flanking region (∗). J Biol Chem. (1996) 271:11911–9. doi: 10.1074/jbc.271.20.11911, 8662618

[68] Sung Y-J Hotchkiss JH Austic RE Dietert RR. L-arginine-dependent production of a reactive nitrogen intermediate by macrophages of a uricotelic species. J Leukoc Biol. (1991) 50:49–56. doi: 10.1002/jlb.50.1.49, 2056246

[69] Orecchioni M Ghosheh Y Pramod A Ley K. Macrophage polarization: different gene signatures in M1(LPS+) vs. classically and M2(LPS−) vs. alternatively activated macrophages. Front Immunol. (2019) 10:1084. doi: 10.3389/fimmu.2019.01084, 31178859 PMC6543837

[70] Sharif O Bolshakov V Raines S Newham P Perkins N. Transcriptional profiling of the LPS induced NF-κB response in macrophages. BMC Immunol. (2007) 8:1. doi: 10.1186/1471-2172-8-1, 17222336 PMC1781469

[71] Cha RS Thilly WG. Specificity, efficiency, and fidelity of PCR. Genome Res. (1993) 3:S18–29. doi: 10.1101/gr.3.3.S18 8118393

[72] Budiarto B Mustopa A Ningrum R Amilia N Saepudin E. Gold nanoparticles (AuNP)-based aptasensor for enteropathogenic Escherichia coli detection. Mol Biol Rep, (2022) 49:9355–63. 35896842 10.1007/s11033-022-07786-3

