# Supplementary material for: In vitro evaluation of CD40-targeting aptamer-based DNA vaccine adjuvants using HD11 cell culture
Source: Front Vet Sci. 2026 Mar 30;13:1735450. doi: 10.3389/fvets.2026.1735450 (PMC13070762; doi:10.3389/fvets.2026.1735450)
Supplement: Supplementary file 1 [file supplementary_file_1.docx]

**Supplementary File**

*In vitro* Evaluation of CD40-Targeting Aptamer-Based DNA Vaccine Adjuvants using HD11 cell culture

Abigeal I. Omolewu^1^, Santiago U. Diaz^1^, Seong W. Kang^1^, Christine N. Vuong^1^,

Adil Al-Ogaili^2^, Billy M. Hargis^1^, Young Min Kwon^1,3*^

**Optimization of the primer concentration for RCA**

Optimizing primer concentration is crucial in nucleic acid amplification such as polymerase chain reaction (PCR) and RCA, as excessively high primer concentrations can lead to nonspecific amplification products and the formation of primer dimers (Cha & Thilly, 1993). In this study, RCA products generated with 0.5 µM and 5 µM primer concentrations were evaluated for their immunostimulatory effect using a nitric oxide assay with HD11 chicken macrophage cell line. The results are summarized in Table S1.

Statistical analysis revealed no significant difference in nitric oxide liberation between RCA products prepared with 0.5 µM and 5 µM primer concentrations. However, a consistent numerical trend was observed across constructs, with RCA products generated using 0.5 µM primers showing slightly lower NO levels compared to those produced with 5 µM primers.

These findings indicate that increasing primer concentration from 0.5 µM to 5 µM does not significantly enhance macrophage activation as similar trends were observed for all the RCA tested. Therefore, using 0.5 µM primers may offer a more cost-effective option for large-scale RCA vaccine production without compromising functional performance.

**Table S1**. Evaluation of the effect of primer concentration (0.5 µM vs. 5 µM) determined by nitric oxide release in a cell line culture assay using HD11 cell.

| **Samples** | **Primer concentration** | **Nitric oxide released**  **(µmol)** | **Statistical grouping**^b^ |
| --- | --- | --- | --- |
| RCA_v3+ST^a^ | 0.5 µmol | 25.61 | b |
| RCV_v3+ST | 5.0 µmol | 26.54 | b |
| Aptamer RCA II | 0.5 µmol | 19.15 | bc |
| Aptamer RCA II | 5.0 µmol | 19.31 | b |
| None | 0.0 µmol | -1.28 | d |
| Mouse IgG | 0.0 µmol | -1.49 | d |
| LPS | 0.0 µmol | 65.19 | a |
|  |  |  |  |

^a^ST (killed *Salmonella* Typhimurium) was used as the antigens.

^b^The letters in the column Statistical Grouping indicate statistical significance, where treatment groups sharing the same letter are not significantly different, and treatment groups not sharing the same letter are significantly different based on one-way ANOVA followed by Tukey's post hoc test (P < 0.05).

**
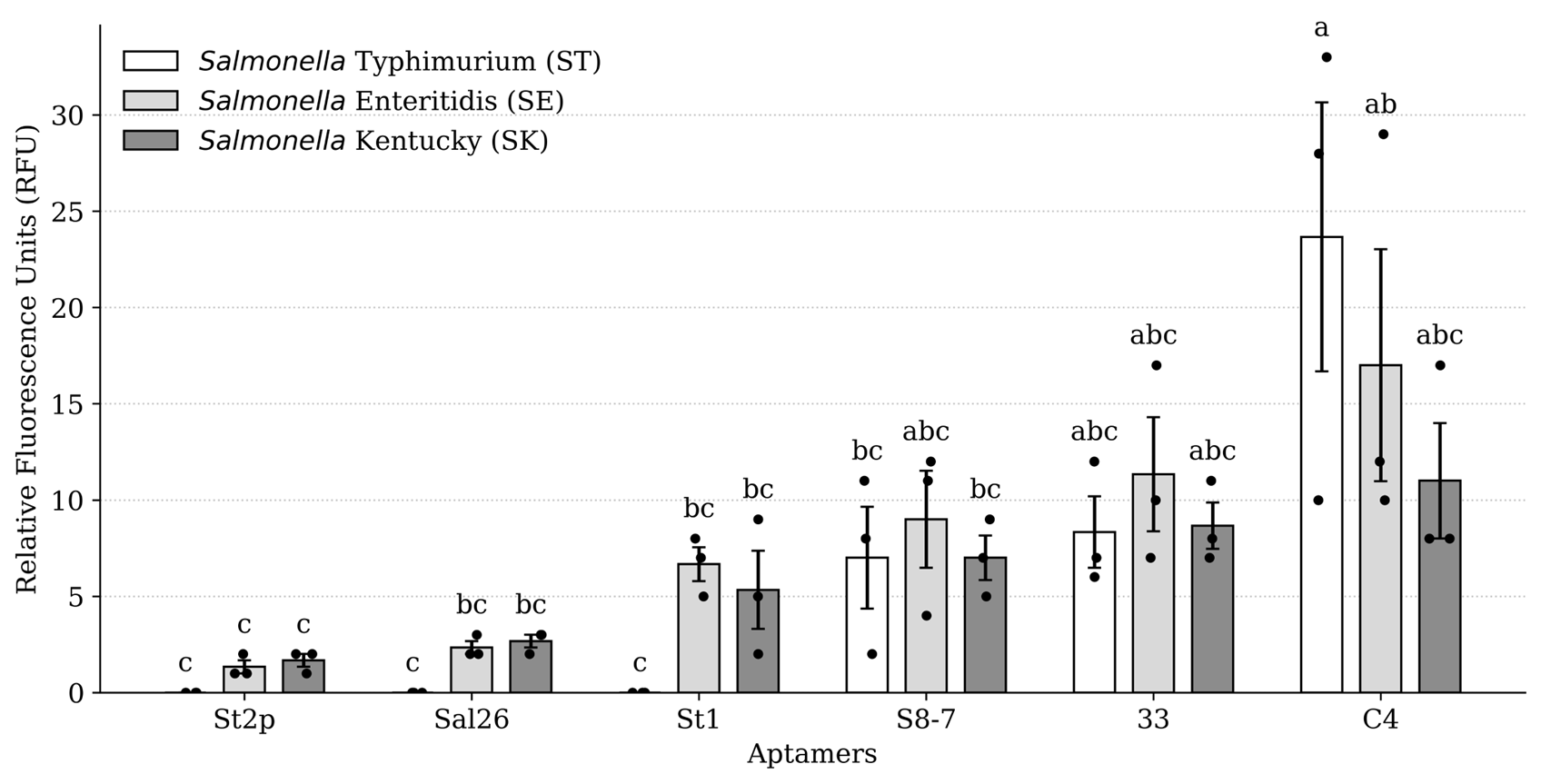
**

**Figure S1**. **Selection of anti-*Salmonella* aptamers.** Binding affinity of six 6-FAM–labeled aptamers (St2p, Sal26, St1, S8-7, 33, and C4) to *Salmonella* Typhimurium, *S.* Enteritidis*,* and *S.* Kentucky. Fluorescence intensity was used as a measure of aptamer binding. Data represent mean ± SD of n = 3 independent biological replicates, each measured in technical triplicate. Different lowercase letters above the bars indicate statistically significant differences among treatments (P < 0.05) as determined by one-way ANOVA followed by Tukey's post hoc test. Bars sharing the same letter are not significantly different (P < 0.05).


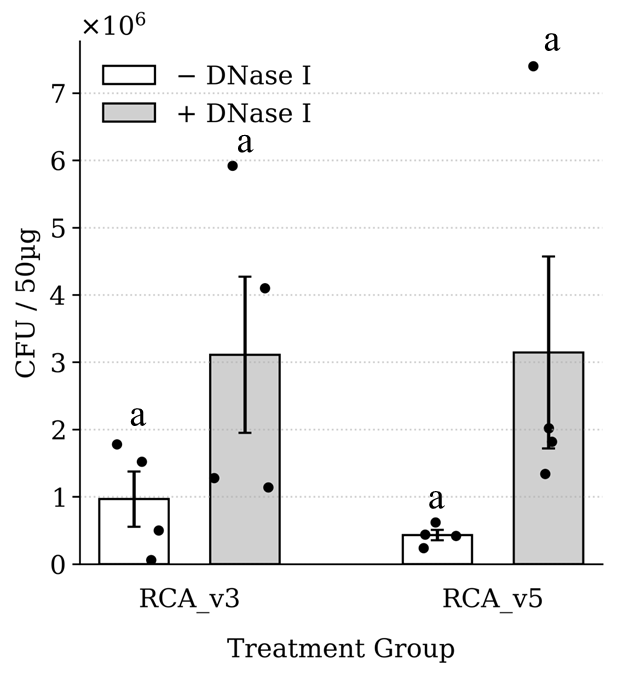


**Figure S2. Quantification of *Salmonella Enteritidis* binding to RCA vaccine constructs.** SE binding to RCA_v3 and RCA_v5 was evaluated using streptavidin-coated magnetic beads. Treatments included two RCA constructs (RCA_v3 and RCA_v5) with (+DNase I) or without (−DNase I) DNase I. These results represent the mean ± standard deviation of 4 independent experiments conducted on separate days using independently prepared samples, without additional technical replication. Bars sharing the same letter are not significantly different (P < 0.05).

The entire original picture of the gel image shown in **Figure 2**
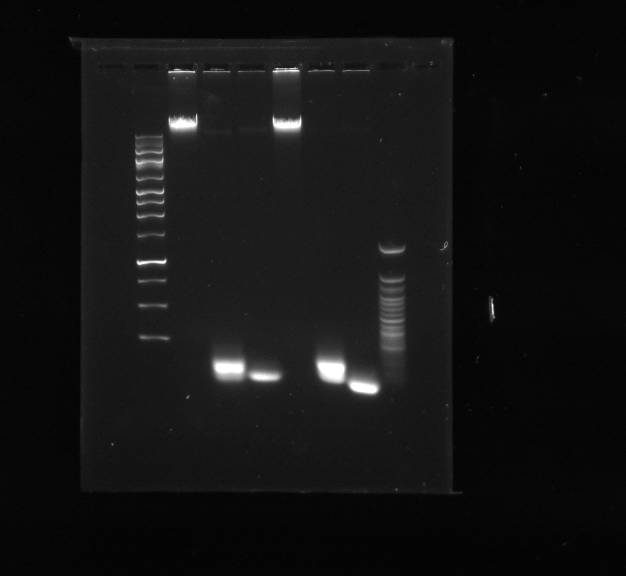


.
